# Supplementary material for: Constitutive hydrogen inhalation prevents vascular remodeling via reduction of oxidative stress
Source: PLoS One. 2020 Apr 17;15(4):e0227582. doi: 10.1371/journal.pone.0227582 (PMC7164592; doi:10.1371/journal.pone.0227582)
Supplement: S1 Table — (DOCX) [file pone.0227582.s002.docx]

**Supplementary Table 1**

| Gene | Primer |
| --- | --- |
| Nox-1 | Forward: 5’-TGGCTAAATCCCATCCAGTC-3’  Reverse: 5’-CCCAAGCTCTCCTGTGTTTG-3’ |
| p40phox | Forward: 5’-TTTGAGCAGCTTCCAGACGA-3’  Reverse: 5’-GGTGAAAGGGCTGTTCTTGC-3’ |
| p47phop | Forward: 5’-GTCCCTGCATCCTATCTGGA-3’  Reverse: 5’-GGGACATCTCGTCCTCTTCA-3’ |
| GAPDH | Forward: 5’-ATGTAGGCCATGAGGTCCAC-3’  Reverse: 5’-TGCGACTTCAACAGCAACTC-3’ |
