# Supplementary figures and images for: Constitutive hydrogen inhalation prevents vascular remodeling via reduction of oxidative stress
Source: PLoS One. 2020 Apr 17;15(4):e0227582. doi: 10.1371/journal.pone.0227582 (PMC7164592; doi:10.1371/journal.pone.0227582)

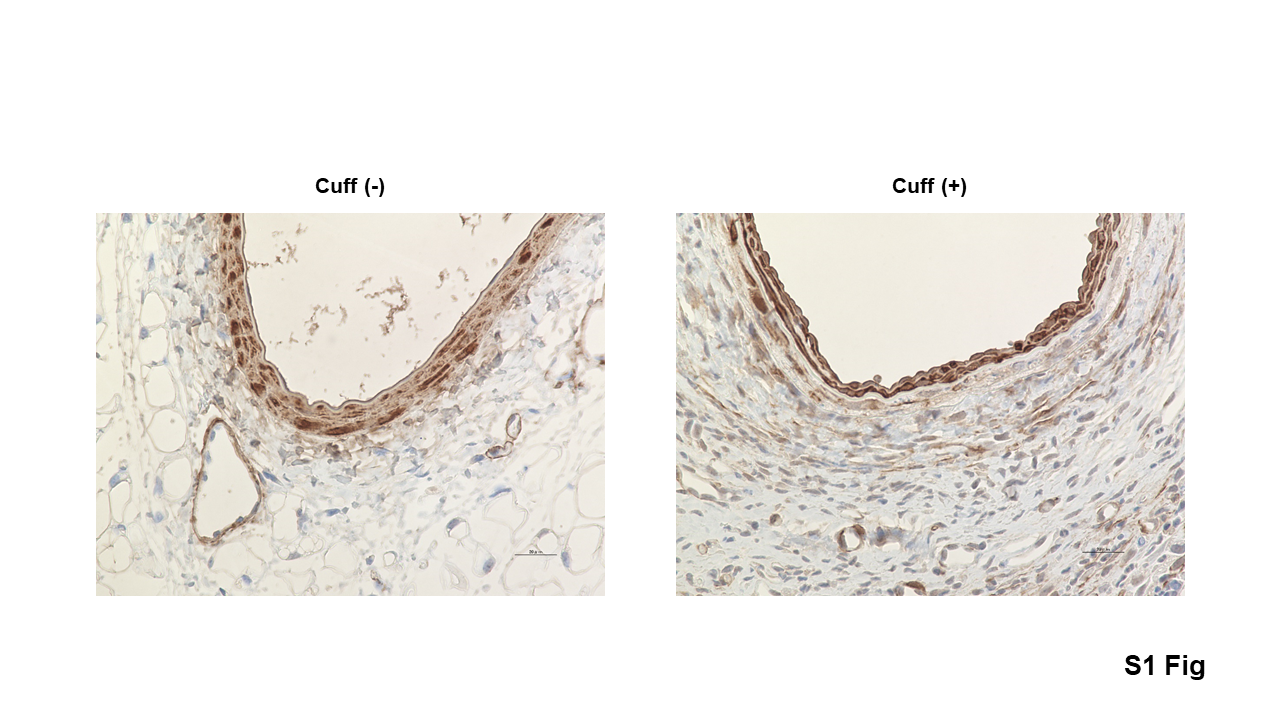

Supplement: S1 Fig — Representative photos of cross-sections of cuff (-) and cuff (+) femoral artery after immunohistochemical staining (for α-SMA). The cuff (+) femoral artery was sampled after 14 days of cuff placement. Sections were stained with the primary antibody, α-SMA antigen antibody (SIGMA, MO, USA). The methods were described the same as above in section of Immunohistochemical Staining (PCNA). Original magnification ×200 (scale bar: 30 μm). (TIF) [file pone.0227582.s003.TIF]

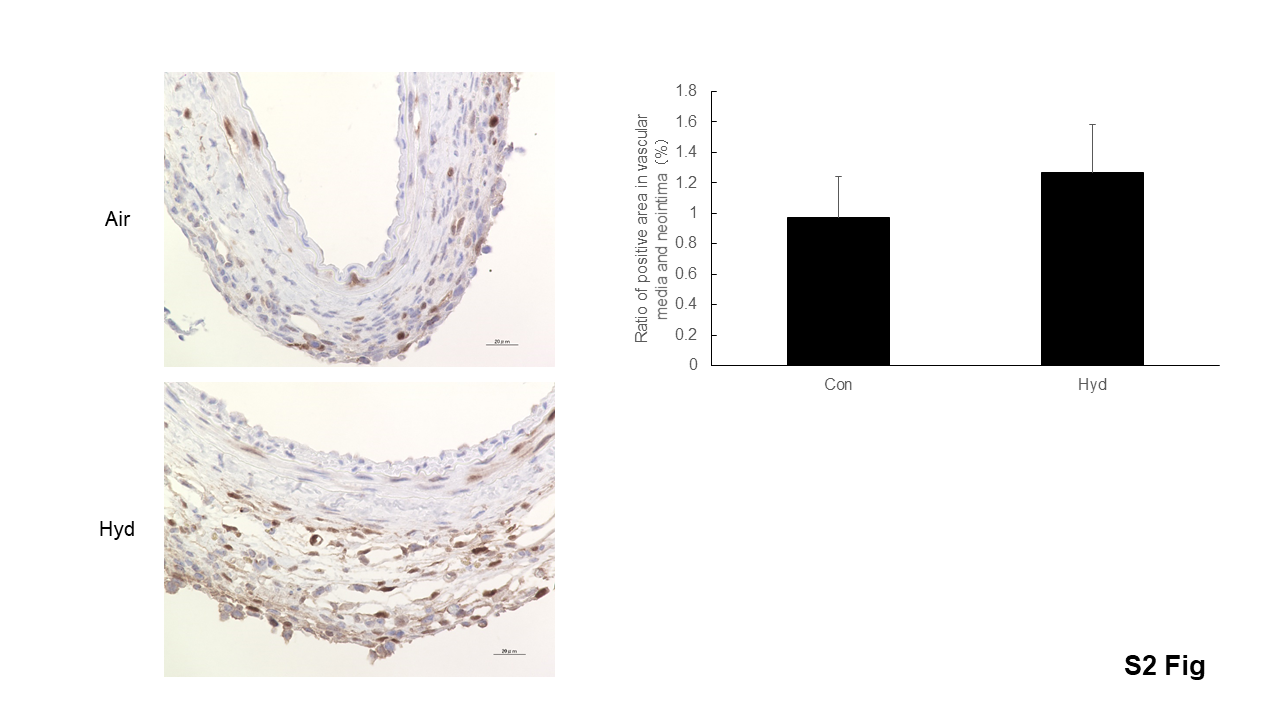

Supplement: S2 Fig — Representative photos and quantitative analysis of cross-sections of injured femoral artery after immunohistochemical staining (for phosphorylated-ERK). Sections were stained with the primary antibody, phosphorylated-ERK antigen antibody (Cell Signaling Technology, MO, USA). The methods were described the same as above in section of Immunohistochemical Staining (PCNA). Original magnification ×200 (scale bar: 30 μm). Original magnification ×600 (scale bar: 20 μm). In quantitative analysis, data represent the ratio of phosphorylated-ERK-positive area in neointima and vascular media, and values are mean ± SEM (n = 16 for air group (Con), n = 15 for hydrogen group (Hyd)). P = 0.48 vs. Con. (TIF) [file pone.0227582.s004.TIF]

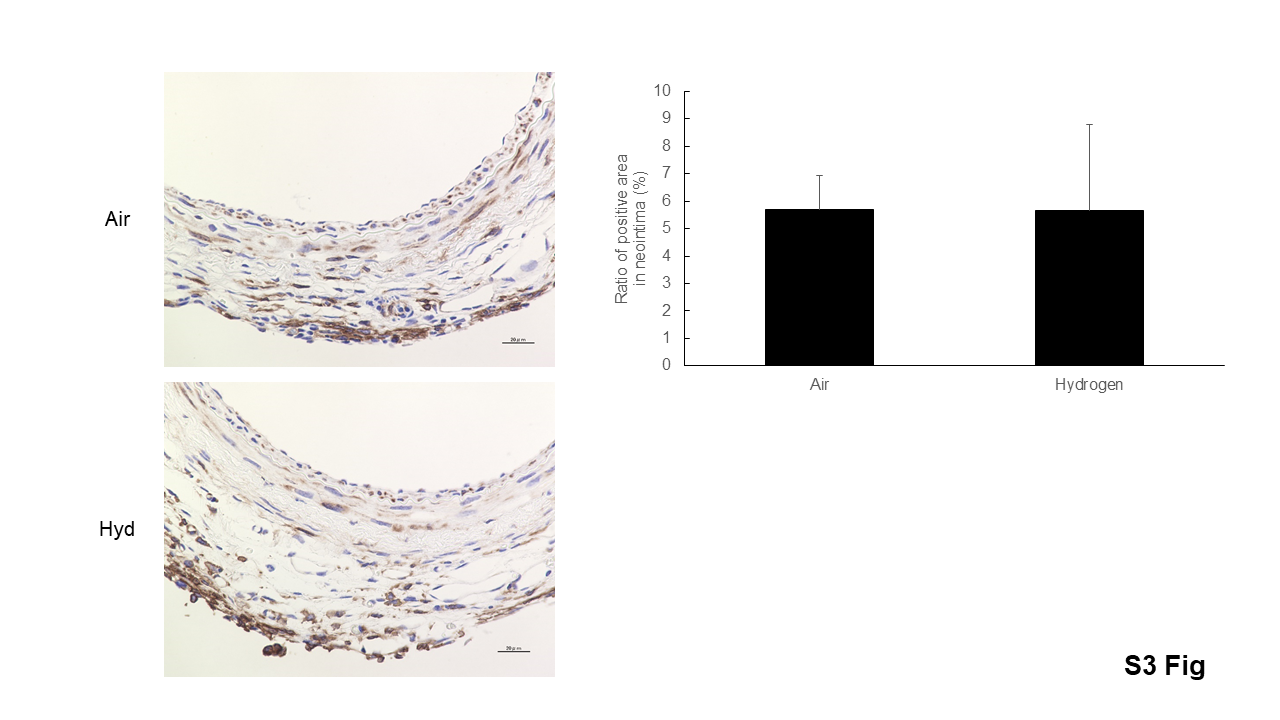

Supplement: S3 Fig — Representative photos and quantitative analysis of cross-sections of injured femoral artery after immunohistochemical staining (for F4/80). Sections were stained with the primary antibody, F4/80 antigen antibody (BMA Biomedicals, Augst, Switzerland). The methods were described the same as above in section of Immunohistochemical Staining (PCNA). Original magnification ×600 (scale bar: 20 μm). In quantitative analysis, data represent the ratio of F4/80-positive area in neointima, and values are mean ± SEM (n = 16 for air group (Con), n = 15 for hydrogen group (Hyd)). P = 0.98 vs. Con. (TIF) [file pone.0227582.s005.TIF]
