# Supplementary material for: Constitutive hydrogen inhalation prevents vascular remodeling via reduction of oxidative stress
Source: PLoS One. 2020 Apr 17;15(4):e0227582. doi: 10.1371/journal.pone.0227582 (PMC7164592; doi:10.1371/journal.pone.0227582)
Supplement: Attachment — Submitted filename: Plos one comments.docx [file pone.0227582.s006.docx]

In this work, the authors investigated the effect of hydrogen gas inhalation to prevent vascular diseases. The authors have demonstrated that constitutive inhalation of hydrogen gas has a beneficial effect on vascular remodeling, partly due to its inhibitory effects on ROS generation, DNA damage and cell proliferation. I think this is a meaningful work drawing some interesting conclusions.

Major comment:

Hydrogen gas is reported to have no known side effects as it neither interferes with metabolic redox reactions nor affects signaling reactive oxygen species. Control group and control rats with hydrogen gas inhalation group must be included in this study to study the protection mechanism of hydrogen gas at a safe concentration.

Minor comments:

1. What does ‘compressed air containing 1.25% hydrogen gas (O2 21%, N2 79%)’ mean?
2. There are many subtypes of NADPH oxidase, NOX 2 and NOX4 has reported to related to oxidative stress. Therefore, the NOX1 is not enough to prove ROS rise in the model.
3. The free radicals, such as O2-, OH-, ONOO- are maintained in the tissue for a relatively short time. After the tissue is processed, these free radicals are likely to degrade. Whether the real free radical signal was detected with the probes of DHE and HPF. The probe of HPF could detect the signals of OH-, ONOO- ? The free radical signal should be detected by ESR (Electron Spin Resonance) or EPR (Electron Paramagnetic Resonance) with Specific probes.
4. The quality of all pictures needs to be improved.
